# The perioperative dynamics of IL-7 following robot-assisted and open colorectal surgery

**DOI:** 10.1038/s41598-018-27245-z

**Published:** 2018-06-14

**Authors:** Małgorzata Krzystek-Korpacka, Marek Zawadzki, Krzysztof Szufnarowski, Iwona Bednarz-Misa, Sabina Gorska, Wojciech Witkiewicz, Andrzej Gamian

**Affiliations:** 1 Department of Medical Biochemistry, Wroclaw Medical University, Wroclaw, Poland; 2 Department of Surgical Oncology, Regional Specialist Hospital, Wroclaw, Poland; 3 Infection Control Unit, Regional Specialist Hospital, Wroclaw, Poland; 4 Laboratory of Medical Microbiology, Ludwik Hirszfeld Institute of Immunology and Experimental Therapy, Polish Academy of Sciences, Wroclaw, Poland; 5 Research and Development Centre at Regional Specialist Hospital, Wroclaw, Poland

## Abstract

Interleukin-7 is critical for T-cell development and displays antimicrobial and antitumor properties. It is referred to as a “critical enhancer of protective immunity”. However, there is no information on interleukin-7 dynamics following colorectal surgery. Moreover, although robot-assisted surgery is gaining popularity, data on the immune response to it is almost non-existent. In this prospective non-randomized case-control study we found interleukin-7 dynamics to differ following robot-assisted and open approach and to affect postoperative immunity. Linear increases were seen in the robotic group while a cubic pattern with a maximum at 8 h in the open one. Low preoperative interleukin-7 was associated with developing surgical site infection. In turn, higher preoperative interleukin-7 was associated with preserved immune function: less pronounced drop in lymphocyte count and higher Δlymphocyte/Δneutrophil ratio in patients undergoing robotic surgery. The changes in other cytokines, namely, interleukin-12(p70), TNFα, interferon-γ, and interleukin-10 were independently associated with interleukin-7 dynamics. In turn, relative changes in interleukin-7 were independent predictors of changes in interferon-γ, key cytokine of favourable Th1 immune response. Taken together, we demonstrated different perioperative dynamics of interleukin-7, which may contribute to favourable outcomes following robotic colorectal surgery including lower incidence of surgical site infections, milder surgery-induced lymphopenia, and beneficial interferon-γ dynamics.

## Introduction

Surgical resection plays a pivotal role in the treatment of colorectal cancer. At the same time, surgical intervention evokes a systemic stress response, particularly to the intestinal tract which is especially susceptible^1^. Surgery, as well as cancer disease itself, shifts immune balance towards unfavourable Th2 immunity. Minimally invasive surgery (MIS) is associated with less surgical stress, quicker recovery, and reduced rates of complication^2,3^. On the cellular and molecular levels, MIS has been linked to better preservation of immune function and attenuated inflammatory response^4^. Robot-assisted colorectal surgery is a step beyond laparoscopy in MIS and has more recently gained momentum, particularly in patients with rectal cancer^5^. Contrary to laparoscopy, however, literature on inflammatory and immune response to robot-assisted surgery is limited.

Interleukin-7 (IL-7) is a pleiotropic cytokine critical for T-cell lymphopoiesis^6–8^. It displays antiviral, antibacterial, antifungal, and antitumor properties^9–13^ and, as such, has been referred to as a “critical enhancer of protective immunity”^14^. In animal models of cancer, IL-7 has been shown to prolong the survival of tumour-bearing hosts^15^. Using IL-7 to boost immunity with IL-7 has been shown to be beneficial in patients with incurable malignancy by preventing disease recurrence and facilitating restoration of immune function^16^. Yet, the issue of IL-7 dynamics following colorectal surgery, either open or MIS, is unknown.

The goal of this study was to analyse the perioperative dynamics of IL-7. We hypothesized that it might be different following robotic and open colorectal surgeries and translate into more favourable outcomes in patients undergoing MIS. Taking into account the biological activity of IL-7, we made an attempt to examine its association with immune function assessed in terms of lymphocyte count, lymphocyte-to-neutrophil ratio, and the dynamics of Th1 cytokine interferon (IFN)-γ as well as the occurrence of surgical site infections.

## Results

From March 2013 to June 2015, 28 patients were enrolled into the open colorectal surgery (OCS) group and 33 into the robot-assisted colorectal surgery (RACS) group. Intraoperative conversions to open procedures occurred in three patients undergoing RACS. For the purpose of the subsequent cytokine analysis, these patients were transferred from the robotic to the open group. Patient demographics and perioperative data is summarized in Table 1.

**Table 1:** Characteristics of study population.

	Open surgery	Robotic surgery	P value
**Size, n**	31	30	
**Patient-associated data:**			
**Sex distribution** [females/males]	14/17	7/23	0.127^a^
**Age** [yrs.]	68 (65–76)	67 (61.5–71.8)	0.302^b^
**Patients’ general physical status** ASA 1/2/3	6/20/5	5/20/5	0.830^c^
**BMI, mean (95%CI)**	26.8 (25.2-28.5)	26.7 (24.9-28.4)	0.872^b^
**HGB, mean (95%CI)**	11.9 (1.2-12.6)	12.3 (11.6-13)	0.456^b^
**Disease-associated data:**			
**CRC stage, n:** stage 0/I/II/III/IV	2/2/15/9/3	2/3/11/12/2	0.839^c^
**Extent of primary tumor, n:** Tis/T1/T2/T3/T4	2/1/1/20/7	2/0/5/16/7	0.393^c^
**Lymph node involvement:** N0/N1/N2 (n)	19/4/8	16/8/6	0.395^c^
No of metastatic nodes, median (95%CI)	5 (1.2-7.8)	2.5 (1-8)	0.714^d^
**Distant metastases:** M0/M1	28/3	28/2	0.970^a^
**Grade of differentiation:** G1/G2/G3/G4	3/21/4/1	5/18/5/0	0.519^c^
**Tumor location:** Left colon/right colon/rectum	11/6/14	7/12/11	0.199^c^
**Surgery-associated data:**			
**Length of surgery [min], mean (range)**	137 (50-290)	224 (150-360)	<0.0001^b^
**Total nodes resected, median (95% CI)**	14 (12-17)	13.5 (12-15)	0.700^d^
**Estimated blood loss [ml], median (range)**	200 (30-300)	50 (50-200)	<0.001^d^
**Transfusions, n (%)**	5 (16.1%)	2 (6.7%)	0.425^a^
**Length of stay [days], mean (range)**	7.5 (4-20)	5.8 (4-8)	0.023^b^
**Surgical site infection (SSI), n (%)** superficial/deep/organ-space	10 (32.3%) 3/3/4	2 (6.7%) 2/0/0	0.013^c^
**Complications**^**e**^**, n (%)**	4 (12.9%)	0	0.113^a^

^a^Fisher’s exact test; ^b^*t*-test for independent samples; ^c^χ^2^ test; ^d^Mann-Whitney *U* test; ^e^surgical complications with Clavien-Dindo score ≥ 3.

### Systemic IL-7

**Figure 1:**
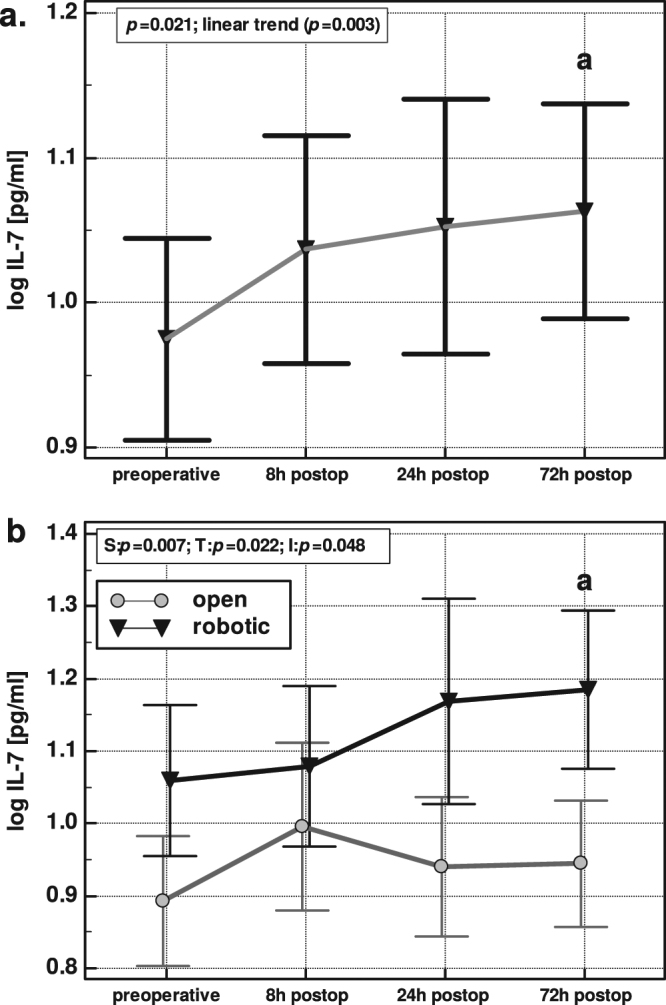
Perioperative dynamics in IL-7 following colorectal surgery (**a**) and the effect of surgical approach (**b**) Data present as geometric means with 95% *CI* and analyzed using repeated measures ANOVA with one- or two-factor design. a, significantly different compare to baseline preoperative level; S, significance of the effect of surgery type; T, significance of the effect of time; I, significance of the interaction between surgery and time.

On average, serum levels of IL-7 increased in response to surgery in a linear manner, with the levels at 72 h post incision significantly higher than at baseline (Fig. 1a). However, as shown in Fig. 1b, postoperative dynamics in IL-7 differed with each surgical approach. Perioperative levels of IL-7 changed significantly with time and differed between the groups. Changes in IL-7 displayed a linear pattern in the RACS group (*P* = 0.003) but a cubic pattern in the OCS group (*P* = 0.044) with a maximum at 8 h post incision.

### Changes in IL-7 in perioperative period

Although both groups were well matched with respect to disease advancement and demographics (Table 1), there was a significant difference in baseline IL-7 levels (means presented on Fig. 1b, *P* = 0.016).

In order to account for baseline differences in IL-7 levels, *t*-test for paired observations was applied. This confirmed significant increases in cytokine levels, both at 8 h post incision compared to baseline in the OCS group (by 1.3-fold on average, *P* = 0.007); and at 72 h post incision compared to baseline in the RACS group (by 1.3-fold, *P* = 0.002). As a result, both surgical strategies were best distinguished by the difference in IL-7 at 8 h and 72 h post incision.

**Figure 2:**
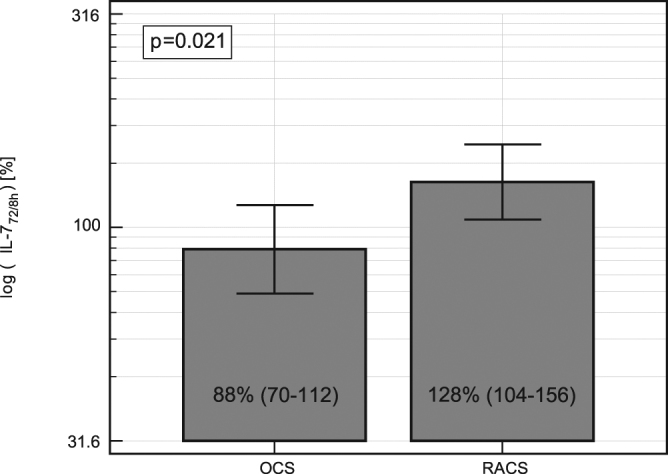
Impact of surgical approach on relative change in IL-7 level between 72 and 8 h post incision (ΔIL-7_72h/8h_). Data present as geometric means with 95% *CI* and analyzed using t-test for paired samples. OCS, open colorectal surgery; RACS, robot-assisted colorectal surgery.

To further verify this observation, a relative change in IL-7 at 72 h expressed as percent of cytokine concentration at 8 h (ΔIL7_72h/8h_ = IL-7_72h_/IL-7_8h_*100%) was calculated and compared between the two groups. At 72 h, IL-7 in the OCS groups dropped by 11.1% but increased by 27.7% in the RACS group, when compared to its levels at 8 h post-incision (Fig. 2).

Since perioperative levels of IL-7 were found related to the incidence of wound infections (analysed in the following sections), two-way ANOVA was applied. The type of surgery (*P* = 0.033), not the occurrence of wound infection (*P* = 0.188), remained a significant factor determining ΔIL7_72h/8h_.

### IL-7 and other cytokines

In order to evaluate to what extent relative changes in IL-7 were accompanied by changes in other pro-inflammatory and anti-inflammatory cytokines, IL-7 correlation pattern was examined (Table 2). In univariate analysis, relative changes in IL-7 and other pro- and anti-inflammatory cytokines were closely interrelated, with the exception of IL-6, although the associations were weaker directly after incision.

**Table 2:** Univariate analysis of correlation pattern between relative changes in IL-7 and IFNγ, TNFα, IL-1β, IL-12(p70), IL-6, and IL-10 at 8, 24, and 72 h post-incision.

	Δ8h/0	Δ24h/0	Δ72h/0
IFNγ	0.61, p < 0.0001	0.82, p < 0.0001	0.8, p < 0.0001
TNFα	0.57, p < 0.0001	0.79, p < 0.0001	0.78, p < 0.0001
IL-12(p70)	0.71, p < 0.0001	0.7, p < 0.0001	0.7, p < 0.0001
IL-6	0.28, p = 0.071	0.20, p = 0.121	0.46, p < 0.001
IL-1β	0.51, p < 0.0001	0.77, p < 0.0001	0.74, p < 0.0001
IL-10	0.69, p < 0.0001	0.71, p < 0.0001	0.86, p < 0.0001

In order to discern independent predictors of relative changes in IL-7 and to determine the partial correlation coefficients, multiple regression (stepwise method) analysis was used and the results are presented in Table 3. A relative increase at 8 h was independently associated with increases in IL-12p70 and IL-10, which explained 47% of ΔIL-7_8h/0_ variability. A relative increase at 24 h was also independently associated with increases in IL-12p70 and TNFα, which explained 70% of ΔIL-7_24h/0_ variability. Finally, a relative increase at 72 h was independently associated with increases in IL-12p70, IFNγ, and IL-10, which explained 82% of ΔIL-7_72h/0_ variability.

**Table 3:** Multiple regression analyses (stepwise method) with relative changes in IL-7 following surgery as dependent variables and respective changes in IFNγ, TNFα, IL-12(p70), IL-6, IL-1β, and IL-10 as explanatory variables.

	Δ8h/0	Δ24h/0	Δ72h/0
	coefficient *b*, r_partial_, *p*	coefficient *b*, r_partial_, *p*	coefficient *b*, r_partial_, *p*
IFNγ	N/I	N/I	*b* = 0.632, *r* = 0.72, *p* < 0.001
TNFα	N/I	*b* = 1.03, *r* = 0.74, *p* < 0.001	N/I
IL-12(p70)	*b* = 0.334, *r* = 0.45, *p* < 0.001	*b* = 0.255, *r* = 0.38, *p* = 0.003	*b* = 0.276, *r* = 0.40, *p* = 0.002
IL-6	N/I	N/I	N/I
IL-1β	N/I	N/I	N/I
IL-10	*b* = 0.128, *r* = 0.30, *p* = 0.022	N/I	*b* = 0.143, *r* = 0.35, *p* = 0.007
R^2^_adjusted_/r_multiple_	0.466/0.695	0.70/0.84	0.821/0.911
*F*-ratio, *p*	27.13, *p* < 0.001	72.07, *p* < 0.001	92.53, *p* < 0.001
constant	1.111	−0.549	−0.094

N/I, not included in the model; variables were entered as logarithms.

### Perioperative dynamics of IL-7 and immune function

#### IL-7 and surgical site infections

**Figure 3:**
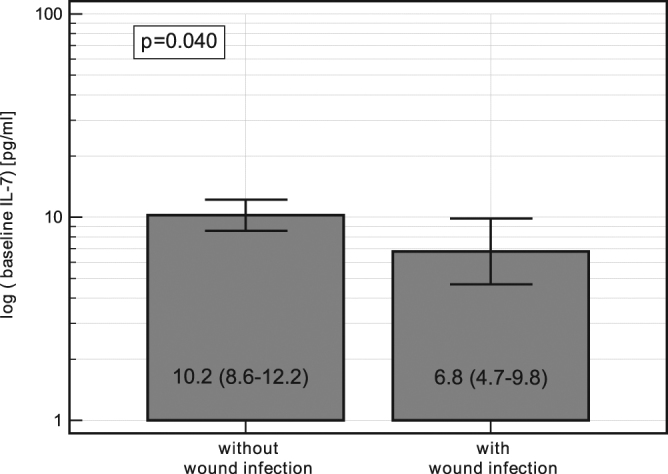
Preoperative IL-7 and occurrence of surgical site infections. Data present as geometric means with 95% *CI* and analyzed using *t*-test for paired samples.

There were no clinical symptoms of surgical site infection (SSI) during the 3-day follow up during which IL-7 was measured. The earliest manifestation of wound infection was on postoperative day 4. Patients developing SSI had lower baseline IL-7 levels than patients without infection (Fig. 3).

The incidence of infection was significantly lower in patients undergoing RACS (Table 1). Relative changes in IL-7 at 72 h compared to baseline (ΔIL-7_72h/0_) was significantly associated with both surgical approaches (*P* = 0.019) and occurrence of infections (*P* = 0.042). The association between ΔIL-7_72h/0_ and infection tended to depend on surgical approach (*P* = 0.083). In fact, both patients who developed infections in the RACS group had more pronounced elevated baseline-adjusted IL-7 at 72 h (ΔIL-7_72h/0_ = 287%) than patients without infection (127%, *P* = 0.018). As a result, there was no significant difference in relative, baseline-adjusted IL-7 at 72 h between patients who developed infection in the OCS group (118%; 131% in patients with deep and organ-space SSI; and 91% in patients with superficial SSI) and those who did not (110%, *P* = 0.767). Both wound infections in the RACS group were superficial as compared to infections in the OCS group, in which superficial SSIs constituted 30% of all wound infections (*P* = 0.079).

#### IL-7 and lymphocyte count and lymphocyte-to-neutrophil ratio

In patients undergoing RACS, the postsurgical drop in lymphocyte count was less pronounced (to 68% of preoperative value (95% CI: 55–76) than in patients undergoing OCS (49% (38–63), *P* = 0.039). The higher preoperative IL-7 levels were associated with a less pronounced drop in lymphocyte count in patients undergoing RACS (r = 0.44, *P* = 0.025) and with higher Δlymphocyte/Δneutrophil ratio (r = 0.45, *P* = 0.020). No such association could be found for neutrophil or total white blood counts.

#### IL-7 as an independent predictor of dynamics of Th1 cytokine IFNγ

Increases in IL-7 were paralleled by the elevations of IFNγ and IL-12p70 (Table 2). Using multiple regressions, it was shown that relative changes in IL-7 were independent predictors of changes in IFNγ at each examined time interval. ΔIL-7_8h/0_, ΔIL-7_24h/0_, and ΔIL-7_72h/0_ explained, respectively, the relative increase in IFNγ at 8 h (r_partial_ = 0.28, *P* = 0.033; other included variables: ΔTNFα_8h/0_, ΔIL-1β_8h/0,_ and ΔIL-10_8h/0_; 84% in ΔIFNγ_8h/0_ variability explained), relative increase in IFNγ at 24 h (r_partial_ = 0.31, *P* = 0.018; other included variables: ΔTNFα_24h/0_ and ΔIL-1β_24h/0_; explaining 83% in ΔIFNγ_24h/0_ variability), and relative increase in IFNγ at 72 h (r_partial_ = 0.62, *P* < 0.0001; other included variables: ΔTNFα_72h/0_, ΔIL-6_72h/_0, and ΔIL-12p70_72 h/0_; explaining 89% in ΔIFNγ_72 h/0_ variability).

## Discussion

Despite the central role of IL-7 in innate and adaptive immunity, literature on this cytokine in the context of colorectal cancer is scarce^6–8^. Its increased circulating levels have been observed in patients with lymph node and distant metastases as well as in individuals with cancer high-risk conditions such as adenomas and inflammatory bowel disease^17^. To the authors’ knowledge, this is the first report to investigate perioperative changes in circulating IL-7 in patients undergoing colorectal resection. Cytokine dynamics were found to differ with respect to the surgical approach. In particular, a steady increase of IL-7 was observed in patients following robot-assisted surgery, while a “rise and drop” pattern was seen following the classic open procedure. There is a good amount of evidence demonstrating differences in the dynamics of cytokines, other than IL-7, following open and laparoscopic colorectal resection which, collectively, show an attenuation of inflammatory response and better preservation of immune function with the laparoscopic approach^4,18^. Interestingly, however, there is no corresponding data concerning the robotic approach. Currently, there is only one study from Shibata and colleagues^19^ that addresses the effect of robot-assisted colorectal surgery on inflammatory and immune responses, by evaluating C-reactive protein and HLA-DR expression on monocytes and lymphocyte subset counts. This pioneering study, however, was conducted on a limited number of patients and the results were highly dispersed, making it difficult to draw reliable conclusions. Only recently, our group showed attenuated inflammatory response following RACS manifesting itself by less pronounced increases in postoperative IL-6 and procalcitonin^20^.

In view of the biological activity attributed to IL-7, its persistent elevation in the early postoperative period following RACS may evoke beneficial immune responses and provide better protection against infection. Surgical trauma causes temporal lymphopenia in the early postoperative period^21^. This phenomenon has been attributed to an increased apoptotic rate through decreased expression of anti-apoptotic Bcl-2^22^. It has also been found to be dependent upon the extent of trauma^23^. Corroborating this notion, Huang *et al*.^18^ reported that patients undergoing MIS had higher postoperative lymphocyte levels than patients undergoing open surgery. The patients in the current study experienced a less pronounced drop in lymphocyte count following RACS than was seen following OCS. Moreover, the decline, as well as the neutrophil-to-lymphocyte ratio in patients undergoing RACS, was tightly correlated with IL-7, which was lower when the preoperative cytokines levels were higher. This observation points to IL-7 function as a major regulator of the number of circulating T cells. IL-7 acts both as a mitogen^6^ and as a survival factor, up-regulating expression of Bcl-2, and down-regulating that of pro-apoptotic Bax and Bim^24,25^.

One of the adverse effects of surgery-induced lymphopenia is increased susceptibility to infections^22^. Accordingly, the incidence of postsurgical infections has been reported to be lower after MIS^26^. Similar to lymphopenia, IL-7 has also been effective in the prevention of infections. In fact, experimental data show that this cytokine protects against infections of viral, bacterial, and fungal origin, specifically during the first week after surgery^9–11,14^. In animal models of fungal and bacterial sepsis^9,10^, treatment with IL-7 improved survival of septic animals by increasing splenic counts, proliferation and activation of CD4+ and CD8+ lymphocytes, as well as by reversing the T-cell defect in cytokine production^9^. As with surgery, sepsis also causes an unfavourable shift from Th1 to Th2 immune response, which manifests itself in part by reduced production of IFNγ^27^. Unsinger *et al*.^9,10^ showed that treatment of septic animals with IL-7 was effective in recovering their IFNγ production. Of note, in current study we observed relatively high SSI rates in OCS group (30%), likely as a result of rigorous SSI surveillance. However, comparable and higher SSI rates after elective colorectal surgery have been reported previously^28–30^.

Recently, Zhang *et al*.^14^ evaluated IL-7 in the model of infection with the enteric rodent pathogen *Citrobacter rodentium*. The authors reported that IL-7 signalling was necessary for tissue recruitment and activation of macrophages. In turn, White *et al*.^31^ evaluated IL-7 in a clinical setting and showed that patients who developed sepsis postoperatively had deficient expression of IL-7 in peripheral blood leukocytes. Likewise, in this current study, the incidence of surgical site infections was found to be lower in the RACS group and the degree of infection less severe. Also consistent with the observations of White *et al*.^31^ and protective role attributed to IL-7, the SSI occurrence was associated with lower cytokine levels prior to surgery.

Surgery-induced immunosuppression also manifests itself by altered T-cell distribution, namely, predominance of immunosuppressive regulatory T-cells and a drop in helper and effector T-cells^32^. It has been repeatedly shown that the imbalance is more profound following open than laparoscopic surgery^32^. The current study showed continuous elevation of IL-7 following the MIS approach, as opposed to its prompt normalization following the open approach, which may serve to provide an explanation of this phenomenon.

Experimentally, recombinant IL-7 increases the percentage of proliferating T-cells by 10-fold. More importantly, the expansion induced by IL-7 is selective. Cytokine preferentially increases the number of recent thymic emigrants, naïve and central memory T-cells, while the expansion of regulatory T-cells is negligible, rendering their percentage in total population relatively decreased^6^. IL-7 also reduces the percentage of senescent T-cells, either by marginally inducing their proliferation compared to other subpopulations^6^, or by protecting against tumour-induced senescence, and thus, dysfunction^33^.

The early postoperative period is a time of considerable immune vulnerability that can facilitate the growth of dormant micrometastases and circulating tumour cells, which can then contribute to disease relapse following curative resection of the primary tumour^34,35^. In this case, persistent elevation of IL-7 observed following RACS may also be beneficial by providing antitumor protection. IL-7 boosts antitumor immunological response via several mechanisms, including promotion of tumour-redirected cytotoxic T lymphocytes, increase of tumouricidal activity in monocytes, or diminishing TGFβ production, and thus weakening tumour-induced suppression of local immune responses^7,12^.

Experimental models have been developed in which cells from the tumour microenvironment were genetically altered to overexpress IL-7^7^. The manipulation resulted in the induction of antitumor immunity and allowed for the eradication of existing tumours as well as for resisting a re-challenge. Hence, in the light of these experimental data, persistent elevation of IL-7 observed after RACS may lessen the immunological vulnerability of cancer patients in the perioperative period. To substantiate this notion, the current study examined if and how relative changes in IL-7 affected dynamics of IFNγ, another cytokine that plays a pivotal role in antitumor, antiviral, and antimicrobial immunity^36^.

Cancer is associated with the suppression of Th1 responses and, as a result, IFNγ is further aggravated by surgical stress^27,34^. A less pronounced disturbance of Th1/Th2 balance is frequently listed among the benefits of MIS^34^. Indeed, increased IFNγ secretion compared to open procedures has been reported for both laparoscopic colectomy and laparoscopic gastrectomy^37,38^. Also in line with findings on IL-7’s ability to up-regulate IFNγ^7,9,10^, the current study found the relative changes in IL-7 to be tightly correlated with those of IFNγ during the entire postoperative period. Moreover, IL-7 was one of the independent predictors determining IFNγ dynamics. Consistent with available literature^6,7,14^, IL-7 was correlated with other cytokines, either involved in IL-7 up-regulation, up-regulated by IL-7, or both. However, dynamics of IL-7 was dependent exclusively on TNFα, IL-12(p70), IFNγ, and IL-10.

This study has to be treated as a pilot one and the observed associations need to be confirmed in randomized trials. At least two other limitations have to be mentioned. One is the fact that data on the count of leukocytes and their fractions had been collected retrospectively and not available for all patients. Concerning close IL-7 association with neutrophil/lymphocyte ratio (NLR) it would be of great interest to further explore NLR dynamics as well as its association with cytokines other than IL-7. The other shortcoming is the difference in preoperative IL-7 concentrations despite the fact that both study groups were well matched with respect to patients’ demographics and the stage of disease. In the light of our prior findings on IL-7 being more elevated in right-sided cancers^20^, the possible explanation might be a slight bias towards this location in a robotic group. Nevertheless, the discrepancy in preoperative IL-7 was addressed by analysing relative and not absolute concentrations of the cytokine and as such did not affect the study results and conclusions.

## Conclusion

The present study shows the differences in perioperative dynamics of IL-7 following robot-assisted and open colorectal surgery. The clinical significance of this finding is uncertain but may contribute to favourable outcomes following robot-assisted surgery as showed here by lower incidence of surgical site infections, milder drop in lymphocyte count, and beneficial IFNγ dynamics.

## Materials and Methods

### Study population

This prospective, comparative, non-randomized study stems from research comparing clinical outcomes, inflammatory, immune, and angiogenic responses and homeostasis in colorectal patients following robotic and conventional surgery^20,39^. This research was conducted as part of the project “WROVASC – Integrated Cardiovascular Center”. The study population consisted of unselected patients with histologically confirmed adenocarcinomas of the colon or rectum. Patients were admitted to the Department of Surgical Oncology, Regional Hospital in Wroclaw between 2013 and 2015. Exclusion criteria included those under 18 years of age, the physical status classification system (ASA) >3, emergency surgery, gross metastatic disease, locally advanced cancers not amenable to curative resection, tumours requiring *en bloc* multi-visceral resection, other synchronous malignancies, severe cardiovascular or respiratory disease, severe mental disorders, or immunological diseases requiring systemic administration of corticosteroids.

Routine preoperative workup involved colonoscopy and computed tomography of the abdomen, as well as a pelvic MRI in case of rectal cancer. Each patient was given the choice to undergo eith**e**r open colorectal surgery (OCS) or robot-assisted colorectal surgery (RACS), using the da Vinci^®^ Si surgical system (Intuitive Surgical, Sunnyvale CA, USA), after receiving detailed information from the operating surgeon as to the advantages and disadvantages of each technique.

Initially, 28 patients were enrolled in the OCS group and 33 in the RACS group. Data on patient demographics, comorbidities, perioperative outcomes, and pathology results were recorded prospectively. The physical status of colorectal cancer patients was expressed in accordance with ASA system. The standard clinical pathway was applied to all patients and included mechanical bowel preparation, low molecular weight heparin and perioperative antibiotic prophylaxis. In the first three postoperative days, parenteral opioids were used for pain control. These were gradually replaced with nonsteroidal anti-inflammatory drugs (NSAIDs).

Patients in both study groups were operated under a standard general anaesthesia. Intravenous agents used for induction included propofol, fentanyl, and rocuronium. Anaesthesia was maintained with sevoflurane. Local anaesthesia or epidural was not used. All patients were given nonsteroidal anti-inflammatory (metamisol) before waking up or immediately after the surgery – in the recovery room. The criteria for discharge included tolerance of soft diet and no apparent complaints or complications.

The Clavien-Dindo Classification was used to objectively assess surgical complications^40^. In short, Clavien-Dindo scale focuses on the therapeutic consequences of a complication grading them into five main groups. Grade I complication represents minor deviation from the normal postoperative course and death of patient is classified as grade V.

Surgical site infections (SSI) were reported prospectively for all patients included in the study. SSIs were defined and classified in accordance with Centers for Disease Control and Prevention criteria^41^ as: superficial incisional SSI, deep incisional SSI and organ/space SSI. Data on SSI were recorded during the hospital stay by direct observation of the surgical wound by the surgeon and trained nurse. Post-discharge surveillance was conducted within 30 days following surgery using patient telephone surveys performed by trained infection control personnel.

Blood samples for IL-7 assessment were collected prior to surgery and at 8, 24, and 72 h post-incision. Postoperative blood cell morphology was collected retrospectively and was available for 54 patients, including 28 who underwent OCS and 26 who underwent RACS.

### Ethical approval

The study protocol was approved by the Medical Ethics Committees of Regional Specialist Hospital. The study was conducted in accordance with the 1964 Helsinki declaration and its later amendments. Informed consent was obtained from all individual participants included in the study.

### Analytical methods

Blood was drawn by venepuncture, allowed to clot for 30 minutes, and centrifuged (15 min., 720 × g). Serum was collected, aliquoted, and kept frozen at −80° until examination.

Levels of IL-7 were measured in duplicate or triplicate by means of a flow cytometry-based method utilizing magnetic microspheres conjugated with monoclonal antibodies. The BioPlex 200 platform with HRF (Bio-Rad, Hercules CA, USA) was used, incorporating Luminex xMAP® technology, Bio-Plex Pro™ Human Cytokine, Chemokine, and Growth Factor Magnetic Bead–Based Assays, according to instructions provided by the manufacturer. Standard curves were drawn using 5-PL logistic regression and the data were analysed using BioPlex Manager 6.0 software. All samples collected for a given patient were measured within the same run. An effort was made to arrange the plates so that each run would include patients from the open and robotic groups in equal proportion.

### Statistical analysis

Normality of distribution was tested using χ^2^ test and homogeneity of variances using Levene’s test. Log transformation was used when appropriate. Data are presented as medians or means with 95% confidence interval (*CI*) or range and analysed using the Mann-Whitney *U* test or *t*-test for independent samples with Welch correction if required. Two-way ANOVA was used to co-examine the effect of surgery and wound infection on IL-7. Paired observations were analysed using *t*-test for paired observations. Repeated measures ANOVA, one and two-factor design with Huynh and Feldt estimates of sphericity (corrected by Lecoutre), was used to compare post-surgical dynamics of IL-7. Correlation analysis was conducted using Pearson test (*r*). Frequency analysis was conducted using Fisher’s exact test or χ^2^ test. A stepwise method of multivariate analysis was conducted in order to discern independent predictors of IL-7 and to determine partial correlation coefficients (net correlation with the effects of other variables removed). Criteria for entering and removing variables were *P* < 0.05 and *P* > 0.1, respectively. The goodness of fit test of the model is presented as coefficient of determination adjusted for the number of independent variables in the regression model (R^2^-adjusted). All calculated probabilities were two-tailed and *P*-values ≤ 0.05 were considered statistically significant. The statistical analysis was conducted using MedCalc Statistical Software version 16.1 (MedCalc Software bvba, Ostend, Belgium).

### Data availability

The datasets generated during and/or analysed during the current study are available from the corresponding author on reasonable request.
